# Author Correction: Efficient targeted transgenesis of large donor DNA into multiple mouse genetic backgrounds using bacteriophage Bxb1 integrase

**DOI:** 10.1038/s41598-023-39015-7

**Published:** 2023-07-20

**Authors:** Benjamin E. Low, Vishnu Hosur, Simon Lesbirel, Michael V. Wiles

**Affiliations:** The Jackson Laboratory, 600 Main Street, Bar Harbor, ME 04609 USA

Correction to: *Scientific Reports* 10.1038/s41598-022-09445-w, published online 31 March 2022

The Data Availability section in the original version of this Article was omitted.

It now appears as below:

“The Mus musculus transgenic K18-hACE2_PrlmnJ sequences are available here https://www.ncbi.nlm.nih.gov/nuccore/ON637243 and https://www.ncbi.nlm.nih.gov/nuccore/ON637244.”

The original Article has been corrected.

