# Supplementary material for: Efficient targeted transgenesis of large donor DNA into multiple mouse genetic backgrounds using bacteriophage Bxb1 integrase
Source: Sci Rep. 2022 Mar 31;12:5424. doi: 10.1038/s41598-022-09445-w (PMC8971409; doi:10.1038/s41598-022-09445-w)
Supplement: Supplementary file 1 — Supplementary Table 1. [file 41598_2022_9445_MOESM1_ESM.docx]

| **Supplemental Table 1: Summary of strains developed and available from JAX** | | | | | | | | | |
| --- | --- | --- | --- | --- | --- | --- | --- | --- | --- |
|  | | | | | | | | | |
| **Single Site Landing Pad Strains (RosaBxb-GT) for RMKI** | | | | | | | | | |
| **Source Strain**  **(Stock #)** | | **JAX Stock #** | | | **Full Nomenclature** | | | **Strain Nickname** | |
| 129S1/SvImJ (2448) | | **35029** | | | 129S1/SvImJ-*Gt(ROSA)26Sor^em12Mvw^*/MvwJ | | | 129S.RosaBxb-GT | |
| A/J (646) | | **35275** | | | A/J-*Gt(ROSA)26Sor^em18Mvw^*/MvwJ | | | AJ.RosaBxb-GT | |
| C57BL/6J (664) | | **28573** | | | C57BL/6J-*Gt(ROSA)26Sor^em2Mvw^*/MvwJ | | | B6.RosaBxb-GT | |
| CAST/EiJ (928) | | **35033** | | | CAST/EiJ-*Gt(ROSA)26Sor^em16Mvw^*/MvwJ | | | CAST.RosaBxb-GT | |
| DBA/2J (671) | | **35031** | | | DBA/2J-*Gt(ROSA)26Sor^em14Mvw^*/MvwJ | | | DBA2.RosaBxb-GT | |
| FVB/NJ (1800) | | **35396** | | | FVB/NJ-*Gt(ROSA)26Sor^em20Mvw^*/MvwJ | | | FVB.RosaBxb-GT | |
| NOD/ShiLtJ (1976) | | **35320** | | | NOD/ShiLtJ-*Gt(ROSA)26Sor^em11Mvw^*/MvwJ | | | NOD.RosaBxb-GT | |
| NSG (5557) | | **29294** | | | NOD.Cg-*Gt(ROSA)26Sor^em3Mvw^ Prkdc^scid^ Il2rg^tm1Wjl^*/MvwJ | | | NSG.RosaBxb-GT | |
| NZO/HILtJ (2105) | | **36178** | | | NZO/HlLtJ-*Gt(ROSA)26Sor^em31Mvw^*/MvwJ | | | NZO.RosaBxb-GT | |
| PWK/PhJ (3715) | | **35276** | | | PWK/PhJ-*Gt(ROSA)26Sor^em19Mvw^*/MvwJ | | | PWK.RosaBxb-GT | |
|  | | | | | | | | | |
| **Dual Site Landing Pad Strains (RosaBxb-GT/GA) for RMCE** | | | | | | | | | |
| **Source Strain/s**  **(Stock #)** | | | **JAX Stock #** | | | **Full Nomenclature** | | | **Strain Nickname** |
| B6.RosaBxb-GT (28573) | | | **36152** | | | C57BL/6J-*Gt(ROSA)26Sor^em7Mvw^*/MvwJ | | | B6.RosaBxb-GT/GA |
| B6(Cg)-Tyrc-2J/J (58), B6.RosaBxb-GT (28573) | | | **36153** | | | B6.Cg-*Gt(ROSA)26Sor^em7Mvw^ Tyr^c-2J^*/MvwJ | | | B6A.RosaBxb-GT/GA |
| NSG.RosaBxb-GT (29294) | | | **36151** | | | NOD.Cg-*Gt(ROSA)26Sor^em5Mvw^ Prkdc^scid^ Il2rg^tm1Wjl^*/MvwJ | | | NSG.RosaBxb-GT/GA |
| NOD/ShiLtJ (1976), NSG.RosaBxb-GT (29294) | | | **36181** | | | NOD(Cg)-*Gt(ROSA)26Sor^em5Mvw^*/MvwJ | | | NOD.RosaBxb-GT/GA |
|  | | | | | | | | | |
| **Available Mouse Strains Generated by RMCE** | | | | | | | | | |
| **Source Strain/s**  **(Stock #)** | **JAX Stock #** | | | **Full Nomenclature** | | | **Strain Nickname** | | |
| B6.RosaBxb-GT/GA (36152) | **36363** | | | C57BL/6J-*Gt(ROSA)^26Sorem25(KRT18-ACE2)Mvw^*/MvwNrosJ | | | B6.RMCE[Krt18-ACE2] | | |
| NSG.RosaBxb-GT/GA (36151) | **35893** | | | NOD.Cg-*Gt(ROSA)^26Sorem27(KRT18-ACE2)Mvw^ Prkdc^scid^ Il2rg^tm1Wjl^*/MvwJ | | | NSG.RMCE[Krt18-ACE2] | | |
