# Supplementary material for: Efficient targeted transgenesis of large donor DNA into multiple mouse genetic backgrounds using bacteriophage Bxb1 integrase
Source: Sci Rep. 2022 Mar 31;12:5424. doi: 10.1038/s41598-022-09445-w (PMC8971409; doi:10.1038/s41598-022-09445-w)
Supplement: Supplementary file 2 — Supplementary Table 2. [file 41598_2022_9445_MOESM2_ESM.docx]

**SUPPLEMENTAL TABLE 2: Relevant sequences of Cas9 guides, donor oligonucleotides, primers, and Bxb1 mRNA.**

| **CRISPR-Cas9 Guides** | **Target Sequence (5' 🡪 3')** | **Target** |
| --- | --- | --- |
| #1888 | GTCTTTCTAGAAGATGGG | ROSA26, site of first attP insertion (attP-GT), Tru-guide (18nt) |
| #2254 | GTCTTTAATCTACCTCGA | ROSA26, site of second attP insertion (attP-GA), Tru-guide (18nt) |
| nCATS-1 | AGCTCACAAGACCTTAGGTC | ROSA26, cuts upstream of attP-GT; use for nCATS |
| nCATS-2 | GGAAAAGTCTCCACCGGACG | ROSA26, cuts downstream of attP-GA; use for nCATS |
| nCATS-3 | TAGGGGTGCAGGGTGGAGGA | Mouse Chr2, cuts upstream of ACE2 insertion site (in JAX#34860) |
| nCATS-4 | TAGTACTTGAGGAACCAAGA | Mouse Chr2, cuts downstream of ACE2 insertion site (in JAX#34860) |
| nCATS-5 | AGCATCATTGATACGGCTCC | ACE2_CDS, cuts inside ACE2 transgene (in JAX#34860) |
| nCATS-6 | AGATGTTACTGATGCAATGG | ACE2_CDS, cuts inside ACE2 transgene (in JAX#34860) |

| **Donor Oligos** | **Sequence (5' 🡪 3')** | **Description** |
| --- | --- | --- |
| #1925 | G*G*TTTGTCTGGTCAACCACCGCGGTCTCAGTGGTGTACGGTACAAA*C*C | attP-GT, phosphothioated bases indicated with *, complement to 1926 |
| #1926 | G*G*TTTGTACCGTACACCACTGAGACCGCGGTGGTTGACCAGACAAA*C*C | attP-GT, phosphothioated bases indicated with *, complement to 1925 |
| #1927 | GGTTTGTCTGGTCAACCACCGCGGTCTCAGTGGTGTACGGTACAAACC | attP-GT, complement to 1928 |
| #1928 | GGTTTGTACCGTACACCACTGAGACCGCGGTGGTTGACCAGACAAACC | attP-GT, complement to 1927 |
| #1969 | GAGGACCGCCCTGGGCCTGGGAGAATCCCTTCCCCCTCTTCCCTCGTGATCTGCAACTCCAGTCTTTCTAGAAGATGGTTTGTCTGGTCAACCACCGCGGTCTCAGTGGTGTACGGTACAAACCGGGCGGGAGTCTTCTGGGCAGGCTTAAAGGCTAACCTGGTGTGTGGGCGTTGTCCTGCAGGGGAATTGAACAGGTG | attP-GT, plus 2x76nt homology arms |
| #1970 | G*A*GGACCGCCCTGGGCCTGGGAGAATCCCTTCCCCCTCTTCCCTCGTGATCTGCAACTCCAGTCTTTCTAGAAGATGGTTTGTCTGGTCAACCACCGCGGTCTCAGTGGTGTACGGTACAAACCGGGCGGGAGTCTTCTGGGCAGGCTTAAAGGCTAACCTGGTGTGTGGGCGTTGTCCTGCAGGGGAATTGAACAGG*T*G | attP-GT, plus 2x76nt homology arms, phosphothioated bases indicated with * |
| #2255 | GCAAAACTACAGGTTATTATTGCTTGTGATCCGCCTCGGAGTATTTTCCATCGGGTTTGTCTGGTCAACCACCGCGGACTCAGTGGTGTACGGTACAAACCAGGTAGATTAAAGACATGCTCACCCGAGTT | attP-GA with asymmetrical homology arms |
| #2020 | CCGGGCGCGACTAGCCAGATATCTGGTCTAGAGCTAGCGAATTCGAATTTAAATCGGATCCCCAACCGGTTGG | MCS Upper, duplex with #2021, clone into SBI MN530A-1 cut with XmaI/StuI 🡪 p5071. |
| #2021 | CCAACCGGTTGGGGATCCGATTTAAATTCGAATTCGCTAGCTCTAGACCAGATATCTGGCTAGTCGCGC | MCS Lower, duplex with #2020, clone into SBI MN530A-1 cut with XmaI/StuI 🡪 p5071. |
| #2025 | CTAGACCGGATGATCCTGACGACGGAGACCGCCGTCGTCGACAAGCCGGCCG | Bxb1 attB Upper, use with #2026 to duplex, clone into p5071 cut with XbaI/EcoRI 🡪 p5087 |
| #2026 | AATTCGGCCGGCTTGTCGACGACGGCGGTCTCCGTCGTCAGGATCATCCGGT | Bxb1 attB Lower, use with #2025 to duplex, clone into p5071 cut with XbaI/EcoRI 🡪 p5087 |

| **PCR Primers** | **Sequence (5' 🡪 3')** | **Target** |
| --- | --- | --- |
| #1670 | GCGCAACGCAATTAATGTGAGTTA | RMCE[Krt18-ACE2], PCR4 (OTI-GA), use with #2966 (842bp) |
| #1699 | GTCGCTCTGAGTTGTTATCAGT | ROSA26, upstream of attP-GT, use for genotyping |
| #1703 | CAGCCTCGATTTGTGGTGTA | ROSA26, downstream of attP-GA, use for genotyping |
| #2161 | GGTTTGTCTGGTCAACCACCGCGGT | attP-GT, use for genotyping |
| #2162 | GGTTTGTACCGTACACCACTGAG | attP-GT & attP-GA, use for genotyping |
| #2164 | ATGATCCTGACGACGGAG | attB, attR, use for genotyping |
| #2287 | GCCACCTGACGTCTAAGAAA | RMCE[Krt18-ACE2], PCR4 (OTI-GT), use with #2962 (1067bp) |
| #2962 | TGCTCTGGAGTGGACCTATT | RMCE[Krt18-ACE2], PCR3 (IOL), use with #1699 (795bp)  Also use in PCR4 (OTI-GT) with #2287 (1067bp) |
| #2964 | GGCCGAGAAGTTCTTTGTATCT | RMCE[Krt18-ACE2], PCR2 (TG), use with #2965 (529bp) |
| #2965 | CCCAACTATCTCTCGCTTCATC | RMCE[Krt18-ACE2], PCR2 (TG), use with #2964 (529bp) |
| #2966 | GCTGAGGGTGATTTGGAGATAA | RMCE[Krt18-ACE2], PCR3 (IOR), use with #1703 (1042bp)  Also use in PCR4 (OTI-GA) with #1670 (842bp) |

| **Bxb1 Int CDS: NLS-HA-Bxb1 (Sequence provided to Trilink, from Addgene Plasmid # 51271) 1557bp** |
| --- |
| ATGCCAAAAAAGAAAAGAAAAGTGTATCCCTATGATGTCCCCGATTATGCCGGTTCAAGAGCCCTGGTCGTGATTAGACTGAGCCGAGTGACAGACGCCACCACAAGTCCCGAGAGACAGCTGGAATCATGCCAGCAGCTCTGTGCTCAGCGGGGTTGGGATGTGGTCGGCGTGGCAGAGGATCTGGACGTGAGCGGGGCCGTCGATCCATTCGACAGAAAGAGGAGGCCCAACCTGGCAAGATGGCTCGCTTTCGAGGAACAGCCCTTTGATGTGATCGTCGCCTACAGAGTGGACCGGCTGACCCGCTCAATTCGACATCTCCAGCAGCTGGTGCATTGGGCTGAGGACCACAAGAAACTGGTGGTCAGCGCAACAGAAGCCCACTTCGATACTACCACACCTTTTGCCGCTGTGGTCATCGCACTGATGGGCACTGTGGCCCAGATGGAGCTCGAAGCTATCAAGGAGCGAAACAGGAGCGCAGCCCATTTCAATATTAGGGCCGGTAAATACAGAGGCTCCCTGCCCCCTTGGGGATATCTCCCTACCAGGGTGGATGGGGAGTGGAGACTGGTGCCAGACCCCGTCCAGAGAGAGCGGATTCTGGAAGTGTACCACAGAGTGGTCGATAACCACGAACCACTCCATCTGGTGGCACACGACCTGAATAGACGCGGCGTGCTCTCTCCAAAGGATTATTTTGCTCAGCTGCAGGGAAGAGAGCCACAGGGAAGAGAATGGAGTGCTACTGCACTGAAGAGATCTATGATCAGTGAGGCTATGCTGGGTTACGCAACACTCAATGGCAAAACTGTCCGGGACGATGACGGAGCCCCTCTGGTGAGGGCTGAGCCTATTCTCACCAGAGAGCAGCTCGAAGCTCTGCGGGCAGAACTGGTCAAGACTAGTCGCGCCAAACCTGCCGTGAGCACCCCAAGCCTGCTCCTGAGGGTGCTGTTCTGCGCCGTCTGTGGAGAGCCAGCATACAAGTTTGCCGGCGGAGGGCGCAAACATCCCCGCTATCGATGCAGGAGCATGGGGTTCCCTAAGCACTGTGGAAACGGGACAGTGGCCATGGCTGAGTGGGACGCCTTTTGCGAGGAACAGGTGCTGGATCTCCTGGGTGACGCTGAGCGGCTGGAAAAAGTGTGGGTGGCAGGATCTGACTCCGCTGTGGAGCTGGCAGAAGTCAATGCCGAGCTCGTGGATCTGACTTCCCTCATCGGATCTCCTGCATATAGAGCTGGGTCCCCACAGAGAGAAGCTCTGGACGCACGAATTGCTGCACTCGCTGCTAGACAGGAGGAACTGGAGGGCCTGGAGGCCAGGCCCTCTGGATGGGAGTGGCGAGAAACCGGACAGAGGTTTGGGGATTGGTGGAGGGAGCAGGACACCGCAGCCAAGAACACATGGCTGAGATCCATGAATGTCCGGCTCACATTCGACGTGCGCGGTGGCCTGACTCGAACCATCGATTTTGGCGACCTGCAGGAGTATGAACAGCACCTGAGACTGGGGTCCGTGGTCGAAAGACTGCACACTGGGATGTCCTAG |
